# Inhibitor of Apoptosis Proteins (IAPs) are commonly dysregulated in GIST and can be pharmacologically targeted to enhance the pro-apoptotic activity of imatinib

**DOI:** 10.18632/oncotarget.9159

**Published:** 2016-05-04

**Authors:** Johanna Falkenhorst, Susanne Grunewald, Thomas Mühlenberg, Adrian Marino-Enriquez, Anna-Carina Reis, Christopher Corless, Michael Heinrich, Jürgen Treckmann, Lars Erik Podleska, Martin Schuler, Jonathan Alfred Fletcher, Sebastian Bauer

**Affiliations:** ^1^ Sarcoma Center, Department of Medical Oncology, West German Cancer Center, University Hospital Essen, University Duisburg-Essen, Essen, Germany; ^2^ Department of Pathology and Neuropathology, West German Cancer Center, University Hospital Essen, University Duisburg-Essen, Essen, Germany; ^3^ Department of Surgery, West German Cancer Center, University Hospital Essen, University Duisburg-Essen, Essen, Germany; ^4^ Department of Pathology, Brigham and Women's Hospital, Boston, MA, USA; ^5^ Department of Pathology, Oregon Health and Science University Knight Cancer Institute, Portland, OR, USA; ^6^ Department of Medical Oncology, Oregon Health and Science University Knight Cancer Institute, Portland, OR, USA; ^7^ German Cancer Consortium (DKTK), Heidelberg, Germany

**Keywords:** GIST, inhibitors of apoptosis proteins, LCL161, TL32711, YM155

## Abstract

Gastrointestinal stromal tumors (GIST) exhibit a strong oncogenic dependency on KIT and KIT inhibitors confer long lasting disease stabilization in the majority of patients. Nonetheless, KIT inhibition alone does not cure GIST as a subset of GIST cells evade apoptosis and eventually develop resistance. Inhibitors of Apoptosis Proteins (IAPs) may confer resistance to drug-induced apoptosis. We observed that the mRNA and protein of IAPs XIAP (*BIRC4*) and survivin (*BIRC5*) were highly expressed in primary GIST tumors and cell line models. Amplification of the respective gene loci (*BIRC2, BIRC3, BIRC4, BIRC5*) was detected in 47% of GIST studied by SNP arrays. Whole exome analyses revealed a mutation of *SMAC(DIABLO)* in a heavily pretreated patient. Both, survivin (rank 62-92/11.194 tested proteins) and XIAP (rank 106-557/11.194) were found to be essential proteins for survival in a synthetic lethality screen. Expression of XIAP and survivin decreased upon KIT inhibition and may play a role in KIT-regulated pro-survival signaling. SMAC-mimetic treatment with LCL161 and TL32711 reduced cIAP1 and XIAP expression. Survivin inhibitor YM155 lead to transcriptional repression of *BIRC5/survivin* (YM155) and induced apoptosis. Combinational treatment with KIT inhibitors (imatinib, regorafenib) enhanced the proapoptotic effect. These findings support the combination of KIT inhibition with IAP antagonists in GIST.

## INTRODUCTION

Gastrointestinal stromal tumors (GIST) are among the most commonly diagnosed mesenchymal tumors and are typically characterized by gain of function mutations in receptor tyrosine kinases (RTKs) *KIT* or *PDGFRA* [1, 2]. Treatment with the tyrosine kinase inhibitors (TKIs) imatinib (IM) and sunitinib (SU) has more than tripled the median overall survival. Nonetheless, KIT-inhibitory treatment alone does not cure GIST as most patients eventually progress and die of their disease [3]. Notably, tumor specimens from patients who underwent metastasectomy following objective remission from imatinib frequently feature viable tumor cells [4]. Secondary *KIT* mutations have been shown to confer imatinib resistance but mechanisms that help GIST cells to evade apoptosis despite effective KIT inhibition are not completely understood [5, 6]. Both autophagy and quiescence have been shown to protect GIST cells from apoptosis [7, 8, 9], but the role of Inhibitors of Apoptosis Proteins (IAPs) has not yet been studied in GIST.

IAPs are essential regulators of apoptosis preventing caspase activation or interfering with proapoptotic signaling intermediates, such as SMAC/DIABLO (Second mitochondria-derived activator of caspases) [10]. Cellular IAPs (cIAP1, encoded by *BIRC2* and cIAP2, encoded by *BIRC3*), X-linked IAP (XIAP, encoded by *BIRC4*), and survivin (encoded by *BIRC5*) are frequently overexpressed in tumors, while being absent in most adult differentiated tissues. In many malignancies, including sarcomas, amplification or high expression of XIAP and survivin correlates with poor prognosis [11, 12, 13, 14, 15].

Following the discovery of IAPs and their potential role in cancer survival, several small molecule IAP inhibitors have been developed, some of which are currently being tested in phase I and II clinical trials. Among these, SMAC mimetics and survivin inhibitors have shown the greatest clinical promise to date [16, 17].

We sought to elucidate the physiological and pathogenic role of IAPs in a GIST cellular context and their possible role for TKI treatment resistance.

## RESULTS

### cIAP1, XIAP and survivin are highly expressed in primary GIST and GIST cell lines

**Figure 1:**
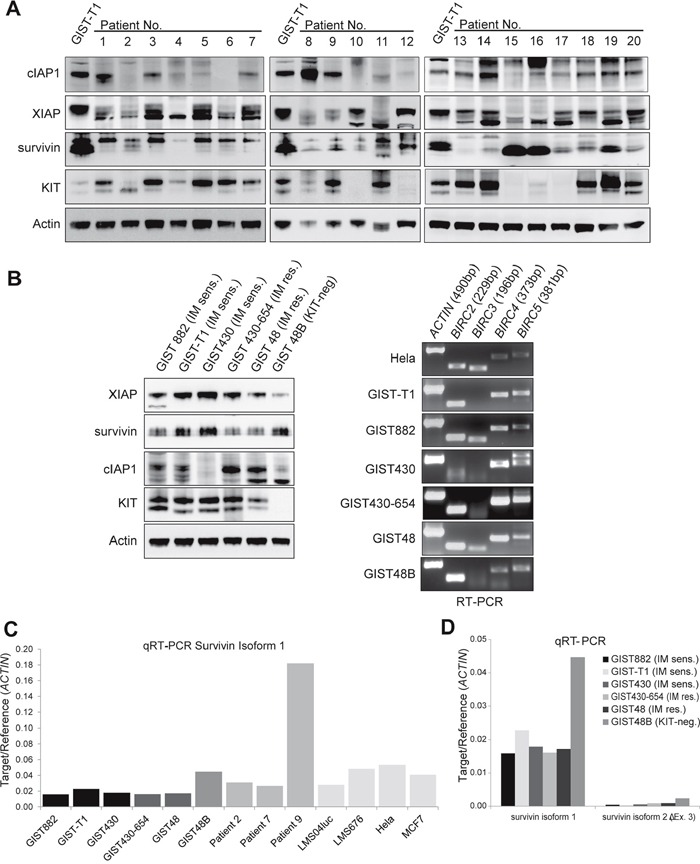
IAP expression in GIST primary tumors and cell lines **A.** Western Blot of 20 GIST primary tumors. Expression of cIAP1, XIAP and survivin was found in 84%, 75% and 80%, respectively and the amount of IAP expression did not correlate with KIT expression levels. (Figure 1A lane 6: no lysate due to sparse tissue sample). **B.** IAP protein and IAP mRNA is expressed in GIST cell lines. Western blot (left panel) and reverse transcriptase PCR (RT-PCR, right panel) show high levels of XIAP and survivin protein expression. IAP mRNA (*BIRC2* (cIAP1), *BIRC3* (cIAP2), *BIRC4* (XIAP), *BIRC5* (survivin)) was detectable in all analyzed cell lines in amounts comparable to positive control (Hela cell line). **C.** Quantitative RT-PCR of survivin isoform 1 in GIST cell lines and primary tumors. Leiomyosarcoma cell lines (LMS04luc and LML676) and Hela and MCF7 cells were included as positive controls to correlate IAP expression levels in GIST. **D.** Quantitative RT-PCR of survivin isoforms 1, 2(*survivin-ΔEx3*) and 3(*survivin-2B*). sens.: sensitive; res.: resistant; No.: number. Data are represented as mean +/− SEM

First, IAP protein expression was examined in 20 GIST tumors and 6 cell lines, using western blot, qRT-PCR and reverse transcriptase PCR (Figure 1). At the protein level, cIAP1 was expressed in 84% (n=19), survivin was expressed in 80%and XIAP in 75% of primary GIST (n=20; Figure 1A) and in all GIST cell lines (Figure 1B). Expression levels were highest in rapidly growing GIST-T1 when compared to the average of primary tumors (XIAP 60% of GIST-T1, survivin 50% of GIST-T1 expression levels; Figure 1A) with exception of cIAP1, where expression levels were comparable to GIST-T1 with variable expression ranging from 27 to 200% of GIST-T1 expression levels. Expression levels of KIT and KIT phosphorylation did not correlate with IAP expression levels (Figure 1A, 1B). IAP expression levels did not correlate with localization or stage of disease (Supplementary Table S1). As we observed varying degrees of survivin expression in independent experiments, we studied how survivin and XIAP levels related to culturing time and confluence. The lowest survivin expression levels were observed 24h after plating at low confluence, with a 1.7-fold increase after 48 hours. Endogeneous apoptosis was highest on day 1 (Supplementary Figure S1). The mRNA and protein of cIAP1 were detectable in all GIST cell lines (Figure 1B). cIAP2 was not evaluable at protein level and RT-PCR revealed low levels of cIAP2 mRNA expression in GIST cell lines. In qRT-PCR experiments, *survivin* mRNA levels (Figure 1C) were lower in the KIT-positive GIST cell lines than in KIT-negative GIST48B. Expression of survivin in GIST48B was similar to the control cell lines Hela and MCF7 [18]. Of note, patient 9, who displayed high mRNA (approx. 5-fold, compared average) and protein levels of survivin was found to have a chromosomal amplification of 17q, containing the survivin gene locus (Figure 1C, Table 1). Patients 2 and 7 had similar levels of survivin mRNA that were 1.6-fold higher than in KIT-positive cell lines. Using qRT-PCR, all cell lines and primary tumors were tested for survivin isoforms 1, 2 (*survivin-ΔEx3*) and 3 (*survivin-2B*). The expression of *survivin-ΔEx3* was 96% lower than isoform 1, whereas *survivin-2B* was not detectable (Figure 1D).

**Table 1:** Frequency of IAP CNVs in GIST tumors

Gene (protein)	Locus	Type of alteration	GEO (25GIST)_a_	Essen (13 GIST)	Total
***BIRC2,3* (cIAP1,2)**	11q22.2	gain	2 (8%)	2 (15.4%)	4 (10.5%)
		loss	3 (12%)	0	3 (7.9%)
***BIRC4* (XIAP)**	Xq25	gain	6 (24%)	2 (15.4%)	8 (21.1%)
***BIRC5* (survivin)**	17q25.3	gain	3 (12%)	3 (23.1%)	6 (15.8%)
		LOH	2 (8%)	0	2 (5.3%)

Abbreviation: LOH: loss of heterozygosity; _a_ GEO dataset GSE20709. SNP array data from 38 GIST tumors was analyzed. 47.4% carried copy number alterations in at least one IAP locus.

### Survivin is the most essential IAP for survival of GIST cells in a lentiviral synthetic lethality screen

**Figure 2:**
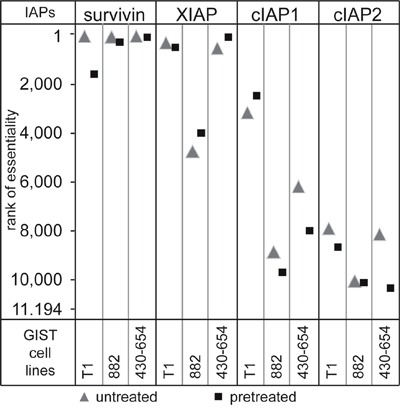
A functional genetic screen of synthetic lethality evaluated the effect of a knockdown of 11,194 proteins on cell proliferation Cells were transfected with a pool of shRNAs and then allowed to proliferate for 6-7 weeks in the presence or absence of KIT inhibition, so that cells with knockdown of essential proteins for proliferation or survival were depleted. Proteins were then ranked for essentiality by their level of depletion.

A synthetic lethality screen featuring 11,194 genes was conducted in GIST-T1, GIST882 and GIST430-654 with and without KIT-inhibitory treatment [19]. Genes were then ranked, with rank 1 signifying the most essential and rank 11,194 the least essential gene for cell proliferation (Figure 2). Survivin was the highest ranking IAP in all untreated cell lines (rank 62-92) and remained important under KIT inhibition in GIST882 and GIST430-654 (ranks 304 and 110, respectively) In GIST-T1, survivin proved less essential under KIT inhibition (rank 1614). XIAP was the second most essential IAP and ranked 106 to 557 in GIST-T1 and GIST430 but not essential in GIST882 (rank 4819). Cellular IAPs were non-essential.

### IAP copy number variations are common events in GIST

Single Nucleotide Polymorphism Array (SNP) data from 38 GIST were analyzed for copy number variations of IAP loci, which could be found in 47.4% of all tumors. 7.9% carried variations in 2 different IAP loci (Table 2, Supplementary Table S2, Supplementary Figure S2). Amplification of IAP loci was found in 15 cases, while loss of heterozygosity (LOH) of the *BIRC2/3* locus was found in 5. In 3 tumors, 2 or more different IAP-associated gene loci were amplified or exhibited LOH of cIAPs. Copy number alterations occurred in patients with localized and metastasized disease. There was no correlation between disease status and IAP alterations. LOH in cIAP genes was observed in patients with localized disease only. In one patient, disease status was unknown (Supplementary Table S2).

### Mutations of negative IAP regulators are rare

Several negative regulators have been described for cIAPs, XIAP and survivin including SMAC/DIABLO, Omi/HTRA2, GSPT1/eRF3, XAF-1, TRAF3 and TP53 [16, 17, 20, 21]. Whole exome sequencing data from 22 patients with high-risk or metastatic GIST (82% of samples were from patients with metastatic disease of whom 61% were resistant to at least IM) were analyzed for presence of inactivating mutations. *TP53* mutations were found in 2 patients and 1 patient had a copy loss. One patient exhibited a frameshift mutation affecting the amino acid T104 of the *SMAC(DIABLO)* gene. No mutations were found for *TRAF3, OMI/HTRA2, XAF1* and *GSPT1/eRF3a*.

### The activity of XIAP and survivin may be co-regulated by KIT signaling

**Figure 3:**
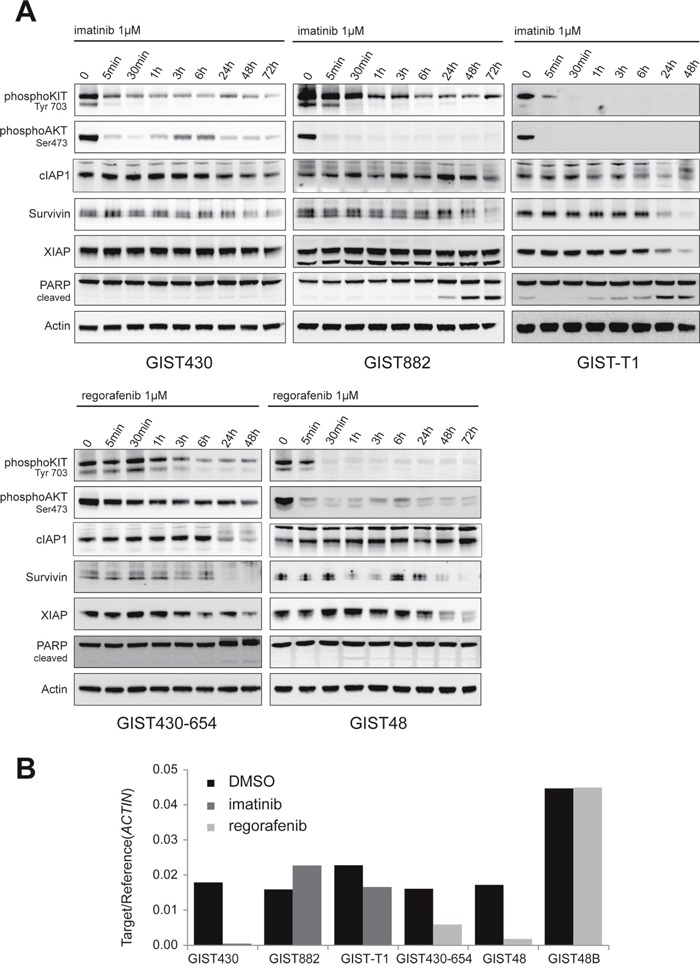
Evaluation of IAP protein and mRNA expression after KIT inhibition **A.** Western Blot time course experiments (0-48/72 hours) in GIST cell lines were conducted with imatinib in imatinib-sensitive and regorafenib in imatinib-resistant cell lines. To assess unspecific effects, KIT-negative GIST48B was treated with imatinib. **B.** Quantitative RT-PCR experiments for survivin were conducted after 48 hours of TKI treatment. KIT inhibition reduces survivin mRNA levels in 4 of 5 cell lines.

To evaluate the influence of KIT signaling on IAP regulation, imatinib-sensitive and imatinib-resistant cell lines were treated with imatinib (1μM) or regorafenib (1μM) for up to 72 hours (Figure 3). To assess KIT-independent cytotoxic effects of imatinib on IAP expression, the KIT-negative cell line GIST48B was treated with imatinib. As expected, strong inhibitory effects on KIT autophosphorylation and KIT signaling intermediates (phospho-AKT) were observed starting as early as 5 minutes. In GIST-T1, GIST430-654 and GIST882 apoptosis, as measured by PARP cleavage, was induced after 3 to 24 hours. XIAP expression decreased in GIST-T1, GIST430-654 and GIST48 but interestingly not in GIST430 and GIST882 (Figure 3A). The expression of cIAP1 protein was not changed by KIT inhibition. Survivin decreased simultaneously in all KIT-positive cell lines after 24-48 hours. In line with these findings, survivin mRNA was markedly reduced in GIST430, GIST430-654 and GIST48 (97, 63 and 90%, respectively), while there was only a 27% reduction in GIST-T1 (Figure 3B). Notably, survivin protein levels showed the latest decrease in GIST882 (72h time point) which was paralleled with an increase of survivin mRNA levels (143%). KIT-inhibitory treatment did not influence IAP protein expression and survivin mRNA in KIT-negative GIST48B (Supplementary Figure S3).

### Survivin inhibitor YM155 has anti-proliferative and pro-apoptotic effects in GIST cell lines

**Figure 4:**
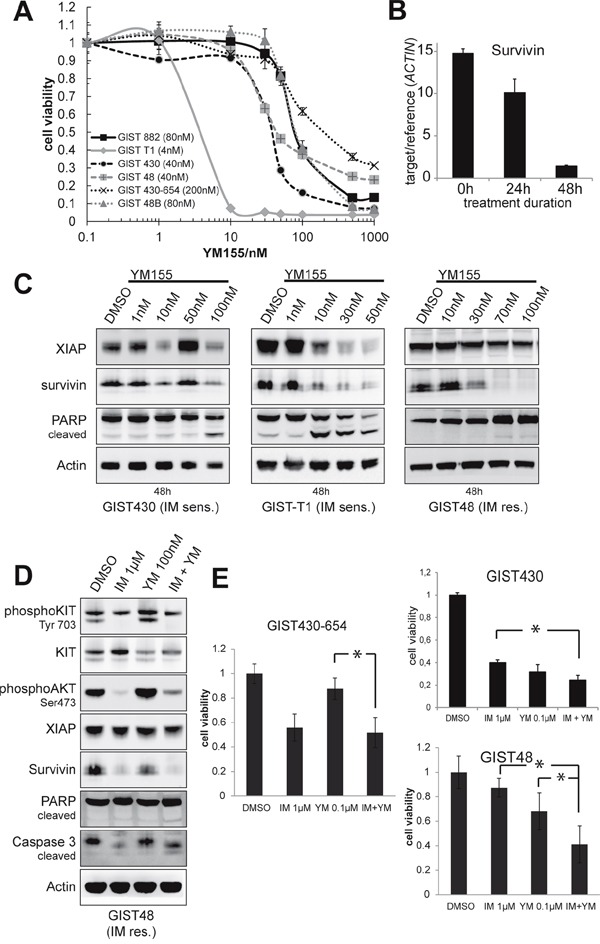
Evaluation of survivin transcriptional repressor YM155 **A.** Cells were treated with increasing doses of YM155 for 6 days and analyzed by SRB viability assay. Data are represented as mean +/− SEM. **B.** qRT-PCR for survivin after treatment of GIST-T1 with 10nM YM155 after 24 and 48 hours. Survivin mRNA levels are decreased in a time-dependent manner with 30% reduction of survivin mRNA at 24h (90% after 48h) of treatment. Data are represented as mean +/− SEM. **C.** Western blot experiments after 48 hours of treatment with YM155 in GIST cell lines. Induction of apoptosis was measured by PARP cleavage. Treatment with YM155 decreases survivin expression at low nanomolar doses. Induction of apoptosis was observed after 48 hours. **D.** Western blot analysis of GIST48 after 48 hours of treatment with YM155 and IM shows a 2.4-fold induction of PARP cleavage compared to DMSO solvent control. **E.** Viability assays after 3 days of treatment with IM, YM and IM+YM in IM-resistant cell lines. Agonistic effects could be observed in GIST430 and GIST48. Data are represented as mean +/− SEM. *: p-value <0,05.

We then sought to investigate the effects of biochemical inhibition of XIAP and survivin on GIST cell lines. YM155 blocks transcription of survivin by interfering with survivin-specific transcription factors [22, 23]. Viability assays with increasing doses of YM155 revealed IC50 values in the low nanomolar scale (5 to 80nM) in all cell lines but GIST430-654 (>1000nM; Figure 4A). Notably, survivin mRNA-levels were reduced by 30% after 24h and 90% after 48 hours of treatment with YM155 10nM in GIST-T1 (Figure 4B). In Western Blot experiments, expression of survivin was inhibited after 24-48 hours of treatment in all examined cell lines (Figure 4C, 4D). Dose-response experiments showed reduction of survivin at low nanomolar doses of YM155 (10-30nM; Figure 4C) and in GIST430 and GIST-T1 concomitant decrease of XIAP could be observed. YM155 induced PARP cleavage in all tested cell lines at concentrations of 10 to 100nM (Figure 4C). Notably, time course experiments with YM155 in GIST-T1 revealed apoptosis preceding the reduction of survivin expression (data not shown). Combined treatment with imatinib (IM) and YM155 had significant agonistic antiproliferative effects in imatinib-resistant GIST48 (p=0,006, compared to single YM and IM), imatinib-resistant GIST430-654 (p<0,001, compared to single YM) and imatinib-sensitive GIST430 (p=0,01 compared to single IM, Figure 4E), whereas only minor increase of apoptosis, as measured by PARP cleavage, was observed (Figure 4D)

### SMAC mimetics downregulate XIAP and induce apoptosis

**Figure 5:**
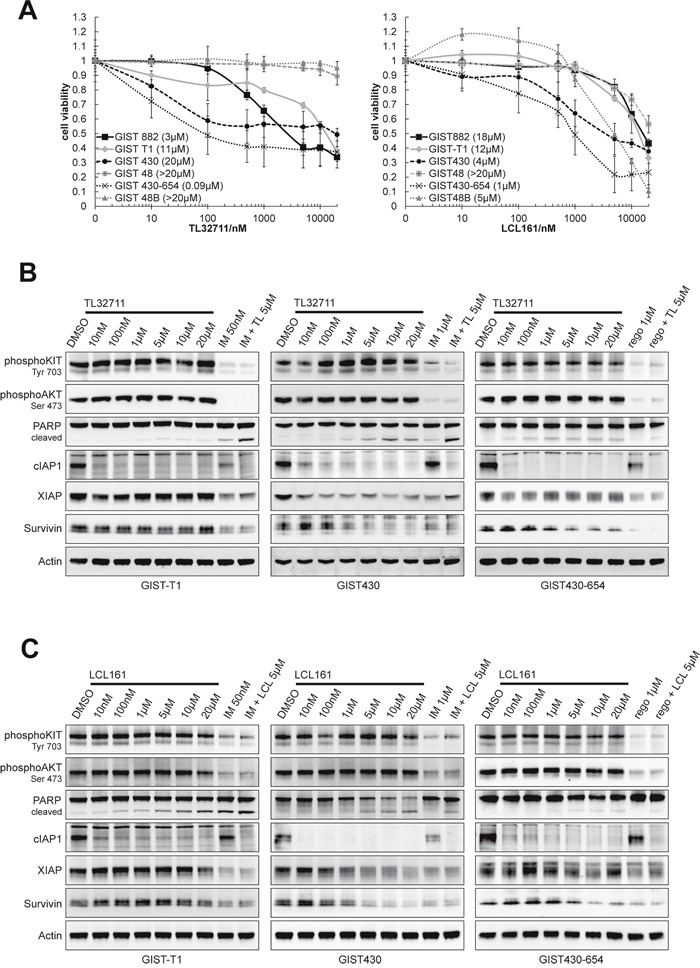
SMAC mimetics TL32711 and LCL161 downregulate XIAP and show agonistic proapoptotic effects when combined with imatinib **A.** Viability assays (SRB) after 6 days of treatment with TL and LCL in GIST cell lines revealed moderate antiproliferative effects. Data are represented as mean +/− SEM. **B-C.** Western Blot following 24 hours of treatment with escalating doses of TL and LCL. A dose of 5μM was combined with KIT inhibitors imatinib (IM) and regorafenib (rego) to evaluate agonistic proapoptotic effects. (B) TL32711 downregulates cIAP1 in all and XIAP in 2 tested cell lines and induces apoptosis in single treatment. Agonistic proapoptotic effect can be observed in combination with imatinib in GIST-T1 and GIST430-11 cell lines. (C) LCL161 downregulates cIAP1 in all cell lines, and reduces XIAP expression in GIST430-11 only. Apoptosis is induced in GIST430-11 and GIST-T1. Agonistic effects with imatinib can be observed in GIST430-11 and GIST-T1.

XIAP and cIAP1 were inhibited by SMAC-mimetic compounds TL32711 (TL) and LCL161 (LCL), which mimic the XIAP- and cIAP-inhibitory interaction of SMAC and IAPs to allow caspase cleavage and apoptosis [24]. Cell viability experiments with TL32711 and LCL161 displayed a differential potency in the GIST cell line panel. After six days of treatment with TL32711, IC50 values from 0.09 (GIST430) to >20μM (GIST48B) were observed (Figure 5A). Treatment with LCL161 resulted in IC50 values from 1 to >20μM with GIST430-654, 430 and GIST48B being the most sensitive cell lines (1, 4, 5μM, respectively, Figure 5A). Combinational treatment with KIT inhibitors and TL32711 showed agonistic antiproliferative effects in GIST-T1, GIST430 and GIST48 (Supplementary Figure S4). Agonistic effects of LCL161 and KIT inhibitors on cell viability could not be observed (data not shown). To assess the proapoptotic effect of single agents TL and LCL and combinations with tyrosine kinase inhibitors, Caspase Glo assays were performed. In GIST-T1 and GIST430, synergistic proapoptotic effects were observed (Supplementary Figure S5). lIn western blot experiments after 24 hours of treatment, cIAP1 expression was reduced by more than 69% (10nM) in all cell lines, whereas XIAP was downregulated by both inhibitors in GIST430 and by TL32711 in GIST430-654 and GIST882 only (Figure 5B–5C, Supplementary Figure S6). LCL161 showed the same effects on XIAP at 100 – 500 fold higher concentrations in GIST430. Survivin was downregulated by the similar concentrations of TL32711 and LCL161 (Figure 5B, 5C). Apoptosis, as measured by cleavage of PARP, was induced in all cell lines tested except in GIST48 and GIST48B. In general, KIT and KIT-dependent signaling (phospho-AKT) was not significantly affected by LCL161 (Figure 5B, 5C). Agonistic induction of PARP cleavage was observed in GIST430 and GIST-T1 (1.4- to 5.5-fold/1.0- to 10.4-fold increase compared to single agent IM and LCL/TL, respectively).

## DISCUSSION

GISTs exhibit a unique oncogenic dependency on mutant KIT which is underscored by the dramatic therapeutic success of the KIT inhibitor imatinib. Nonetheless, KIT inhibition alone is not curative as the majority of patients eventually relapse and complete pathological remissions are rare exceptions. While tumor regression is commonly observed following treatment with imatinib, vital cells are usually found in tumors that have been resected in responding patients [4]. Improving the apoptotic treatment response to imatinib represents a key goal for future therapeutic concepts in GIST. In the CML context, BCL2 inhibitors have been shown to enhance imatinib-induced cell death [25]. Quiescence and autophagy have been demonstrated as potential escape mechanisms to imatinib inhibition in GIST. However, the role of Inhibitors of Apoptosis Proteins (IAPs) has not yet been investigated in GIST. This study is the first to comprehensively investigate the oncogenic and therapeutic relevance of IAPs in GIST.

IAPs promote cell survival by interacting with apoptotic pathways and triggering cell cycle progression. While cIAP1 and cIAP2 inhibit extrinsic apoptosis and activate NF-κB signaling, XIAP directly inhibits effector caspases [16]. In contrast, survivin exhibits indirect anti-apoptotic effects by stabilizing XIAP and inhibiting SMAC [17]. Furthermore, survivin, as part of the chromosomal passenger complex, regulates mitosis at the G2/M checkpoint [17].

The expression of IAPs has previously been studied in the NCI60 cancer cell line panel. In general, cIAP1, XIAP and survivin mRNA and protein were highly expressed in most tumor types. Notably only 50% of cell lines express cIAP2 mRNA and only 5% cIAP2 protein [13]. Consistent with these findings, most GIST expressed cIAP1, XIAP and survivin protein and mRNA while cIAP2 mRNA levels were low. GIST primary tumors and cell lines showed a high concordance of mRNA levels but primary tumors exhibited slightly lower protein expression than cell lines (Figure 1). Of note, survivin protein levels vary substantially in cell lines depending on the confluence of cells (Supplementary Figure S1). Differences in molecular weight of survivin and double bands, most notably in tumor lysates, but also in GIST cell lines, may result from ubiquitinylation of proteins.

It has previously been shown that the loss of survivin triggers mitotic defects and cell death [17], and XIAP-deficient cells are sensitized to radiation-induced apoptosis [26]. Cellular IAPs are not required for cell survival, but the loss of both cIAPs impairs pro-survival signaling (TNFα-mediated NF-κB activation) [27]. In synthetic lethality studies for IAP family proteins using a shRNA screen, survivin, as would be expected due to its essential role in mitosis [16], ranked among the top hundred essential proteins for cell proliferation in untreated GIST cell lines (Figure 2). Notably, KIT inhibition decreased the essentiality of survivin in all three cell lines. This indicates that targeting survivin in GIST may in part depend on the level of proliferation of cells. Apart from survivin, XIAP showed a high essentiality in two out of three cell lines while cIAPs, when knocked-down alone, were not essential regardless of a treatment.

IAP expression is commonly altered in cancer and associated with unfavorable prognosis [11, 12, 13, 14, 15]. Unfortunately the number of low risk, localized GIST was too low to determine whether genomic changes involving IAP family gene loci are early or late events in the oncogenic progression of GIST. The amplification of the survivin locus on 17q has been observed to be more frequent in malignant and metastatic GIST [28] and associated with advanced stages in neuroblastoma [29]. AML patients with *XIAP* amplifications had a shorter overall survival [13]. A *BIRC2/BIRC3* deletion activates non-canonical NF-κB signaling in multiple myeloma [30], whereas 11q22 amplification (containing cIAP locus) was associated with earlier onset of disease in cervical cancer [31]. In our analysis, GIST contained IAP copy number alterations in 47.4% (Table 1). The genetic loci of XIAP and survivin displayed more gains than losses, whereas cIAP loci displayed more losses/LOHs suggesting that in GIST particularly survivin and XIAP may contribute to the transformation of cells. As we observed IAP expression in all GIST cell lines and a majority of GIST tumors, additional factors are most likely contributing to the high IAP expression levels:

Physiologically, XIAP and cIAPs are inhibited following initiation of apoptosis (SMAC/DIABLO, Omi/HTRA2) and ER-stress (GSPT1/eRF3). These inhibitors are physiologically sequestered to mitochondria or the endoplasmic reticulum. The release during ER-stress or apoptosis leads to direct binding of cIAPs and XIAP. The binding prevents further interaction of IAPs e.g. with caspases. [16]. Other negatively regulating proteins are XAF-1, which binds XIAP and was found to be downregulated in a majority of the NCI60 cancer cell line panel, and TRAF3, which promotes the degradation of the cIAP1/2-TRAF2-complex [16]. Tumor suppressor p53 downregulates survivin [32] and promotes the mitochondrial release of SMAC [21]. Genetic changes in GIST were only observed in *TP53* and *SMAC/DIABLO*. Interestingly, patient 9 (Figure 1, Supplementary Table S3 (Essen_3)) carried a SMAC/DIABLO mutation. In addition, the patient was found to have a 17q amplification, which resulted in highly elevated survivin mRNA and protein levels. This patient suffered from a highly imatinib-resistant, aggressive, metastatic GIST. However, the low number of samples analyzed in this study does not allow conclusions on the prognostic or predictive relevance of these changes.

To our knowledge, the impact of oncogenic KIT signaling on IAP regulation has yet not been studied in GIST. AKT, which is strongly activated by oncogenic KIT, was found to stabilize XIAP in AKT-transfected human ovarian cancer epithelial cell line A2780S and human embryonic kidney (HEK) 293 cells [33]. The specific inhibition of PDGFRA, a receptor tyrosine kinase closely related to KIT, induced apoptosis in a hypereosinophilic syndrome (HES) cell line but did not influence on IAP expression [34]. Our time course experiments do not clearly support the hypothesis that IAPs play a role in KIT-regulated pro-survival signaling. Expression of survivin and XIAP decreased upon KIT inhibition in the majority of cell lines – however, this decrease was observed as early as 24h and may also just represent an indirect effect of decreased proliferation (in the case of survivin, Figure 3). cIAP1 expression was not affected by KIT inhibition, which underscores the results of our synthetic lethality screen that it may not play a functional role in the GIST cellular context. We also assessed the effect of imatinib on IAPs in a KIT-negative cell line to exclude the possibility of KIT-independent effects of imatinib on IAP expression, which were not found (Supplementary Figure S3). Additional functional studies using lentiviral expression vectors for IAPs are needed to confirm the effect of constitutive IAP overexpression on apoptotic resistance in GIST.

Several biochemical inhibitors of IAPs have been developed and are currently undergoing early clinical investigations. Among these, the survivin transcriptional repressor YM155 and SMAC mimetics like TL32711 and LCL161 are in advanced clinical development [24]. We found that IAP inhibitors may improve the apoptotic response of KIT inhibitors. YM155 has been shown to inhibit tumor growth *in vitro* and *in vivo* in different cancers [35]. Its steady state plasma levels reach 17-32nM (6-day-continuous infusion) with 20-fold higher concentration in mouse xenograft tumors of prostate cancer [36, 35]. YM155 does not directly bind the survivin promoter, but binds survivin-regulating transcription factors [37], which may lead to additional, unspecific effects, e.g. Mcl-1 downregulation [38] and EGFR suppression [39]. In GIST cell lines, target inhibition as measured by survivin mRNA and protein levels was observed at low nanomolar doses and accompanied by decreased cell viability. However, the sensitivity varied between cell lines and we did not find IAP expression levels to be predictive (Figure 4). In time course experiments, the reduction of survivin protein occurred in parallel and not prior to apoptosis (data not shown). These observations suggest that YM155 effects in GIST may involve a variety of mechanisms other than downregulation of survivin among which DNA damage has been described by other groups [40].

We further validated SMAC-mimetic compounds, LCL161 and TL32711, which mimic the IAP-inhibitory interaction of SMAC and cIAPs/XIAP (Figure 5). In phase I studies, the maximum tolerated dose was 4μM for LCL161 and 37μM for TL32711, well within the dose range presented in the studies herein [41]. Disease stabilizations and even partial responses have already been observed in clinical trials (TL32711: CRC and melanoma, LCL161: objective responses with combinational therapy only) [24] As observed by other groups [42, 43], cIAP was also downregulated at low nanomolar doses in our experiments and accompanied by downregulation of XIAP in 3 out of 6 cell lines. Both compounds exhibited pro-apoptotic effects at doses that seem therapeutically achievable and this effect was more pronounced when combined with KIT inhibitors. Notably, downregulation of cIAP1 “alone” was not predictive of response (Figure 4B–4C) which may be explained by the high ranks of cIAPs found in the synthetic lethality screens (Figure 1C). Additional studies might investigate IAP-independent predictors of response for SMAC-mimetics such as TNF-α, which has been shown to sensitize melanoma cells to LCL161 [43], as differential levels of TNF-α have already been observed in GIST [44].

In summary, we found strong evidence that IAPs may co-mediate the pro-survival signaling of oncogenic KIT with survivin and XIAP being the most essential IAP family members. Genetic changes involving IAP gene loci are common in GIST and may independently add to the apoptotic resistance of GIST. The pharmaceutical inhibition of IAPs may improve the apoptotic response of KIT inhibitors. Our studies therefore provide a strong rationale for further investigation of IAP inhibitors as a therapeutic strategy in GIST.

## MATERIALS AND METHODS

### Cell lines, reagents and antibodies

GIST cell lines established from human GIST have been described previously [45]. GIST-T1 was kindly provided by Takahiro Taguchi, Kochi University, Japan [46]. GIST cell lines GIST-T1, GIST882 and GIST 430 are imatinib-sensitive, GIST cell lines GIST48 and GIST430-654 contain secondary KIT resistance mutations and are resistant to imatinib. GIST 48B is a subclone of GIST48 that still harbors the activating *KIT* mutation but does not express detectable KIT transcript or KIT protein. LMS676 and LMS04luc were established from two leiomyosarcomas [47].

A list of cell lines, reagents and antibodies is provided in the Supplementary Material (Supplementary Table S3). LCL161 was kindly provided by Novartis.

### *In vitro* assays

For Sulforhodamine B (SRB) assays [48] cells were plated at 1.000-30.000 cells/well in a 96-well flat bottom plate and cultured for 24 hours. Cells were then incubated with media containing inhibitors or solvent control (DMSO). After 24-144 hours cells were fixed and stained with SRB (0.4%, Sigma-Aldrich, St. Louis, MO, USA) and absorption was measured with a Genion luminometer (Tecan, Crailsheim, Germany). All measurements were carried out in triplicate/quadruplicate wells for at least two times and a representative example is shown.

### Western blotting

Cells were seeded into 6-well flat bottom plates and grown to confluence and then incubated with inhibitors or solvent control. Protein lysates were prepared according to standard protocols [49]. SDS-PAGE was carried out as previously described [50]. Proteins were transferred to Hybond P membranes (Amersham Pharmacia Biotech, Uppsala, Sweden). Beta-Actin was used as a loading control. Changes in protein expression and phosphorylation as visualized by chemiluminescence were captured and quantified using a FUJI LAS3000 system with Science Lab 2001 ImageGauge 4.0 software (Fujifilm Medial Systems, Stanford, CT, USA).

### Caspase Glo ^®^ 3/7 assay

Cells were plated and cultured as for SRB assay. After 24 – 48 hours of incubation, Caspase Glo ^®^ (Promega, Madison, Wisconsin, USA) was added to the media and incubated according to manufacturer's protocol. Luminescence was measured with a Genion luminometer (Tecan, Crailsheim, Germany). All measurements were carried out in triplicate/quadruplicate wells for at least two times and a representative example is shown.

### Reverse transcriptase PCR and primers

Cells were harvested with RNA-Protect (Qiagen). RNA was isolated according to manufacturer's protocols (RNeasy Mini Kit, Qiagen, Hilden, Germany). cDNA synthesis and RT-PCR was done as described previously ([51], see Table 2 for primer sequences).

**Table 2:** RT-PCR primer sequences

Gene (protein)	NM code	Forward primer sequence	Reverse primer sequence
***BIRC2* (cIAP1)**	NM_001166	5-GCT CAG TAA CTG GGA ACC AAA-3	5-ATC ATT GCG ACC CAC ATA ATA-3
***BIRC3* (cIAP2)**	NM_001165	5-GAT GTT TCA GAT CTA CCA GTG-3	5-GAA ATG TAC GAA CTG TAC CCT-3
***BIRC4* (XIAP)**	NM_001167	5-GCA GAT CTA GTG AAT GCT CAG AAA-3	5-TAC TTG GTA GCA AAT GCT AAT GGA-3
***BIRC5* (survivin)**	NM_001168	5-CCG ACG TTG CCC CCT GC-3	5-TCG ATG GCA CGG CGC AC-3

### Quantitative real-time PCR (qRT-PCR)

The cDNA synthesis and qRT-PCR were conducted as described previously [51]. FAM-labeled probes for *KIT* (HS00174029_m1), *BIRC5* (*survivin*) isoform1 (HS00977612_mH), isoform 2 (*survivin-ΔEx.3*; HS03043576_m1), isoform 3 (*survivin-2B*; HS03043575_m1) were purchased from Applied Biosystems (Waltham, MA, USA). Expression levels of the housekeeping gene *β-ACTIN* (hs99999903_m1) were assessed for normalization.

### Functional genetic screen of synthetic lethality

The screening method has been described previously [19]. In GIST-T1, GIST882 and GIST430-654 cells, 11 194 genes were knocked down by lentiviral infection with a pool of 54 020 shRNAs (approx. 5 shRNAs per gene). After puromycin selection, cells were allowed to proliferate independently for 6-7 weeks and treated with imatinib (GIST882 and GIST-T1) or sunitinib (GIST430-654). Genomic DNA of treated and untreated cells was then isolated for shRNA amplification and massive parallel sequencing. The 54020 shRNAs were then ranked by their relative depletion from the cell pool and the corresponding genes scored according to the rank of the second-most depleted shRNA using the GENE-E program (http://www.broadinstitute.org/cancer/software/GENE-E/download.html).

### SNP 6.0 and 250K array data acquisition and analysis

DNA extraction, 250K Array data acquisition and analysis of 13 GIST tumor specimen was conducted as previously described [51]. In addition, data from previously published GIST (SNP 6.0 array [52]) were extracted from Gene Expression Omnibus (GEO, GSE20709) and further analyzed. This group consisted of 18 metastatic and 13 localized GIST, the clinical stage of 7 other GIST was unknown (Supplementary Table S2). Out of these, 24 were *KIT*-mutated, 7 *PDGFRA*-mutated and 6 wildtype for *KIT* and *PDGFRA* (1 unknown). Raw data was analyzed for copy number variations in IAP loci (cIAP1*(BIRC2)*, cIAP2*(BIRC3)*: 11q22; XIAP(*BIRC4*): Xq25; survivin(*BIRC5*): 17q25), using the Chromosome Analysis Suite 2.0.0.195 (Affymetrix) and CNAG Version 2.0 (Cancer Genomics Project, University of Tokyo, Tokyo, Japan)).

### Next-generation sequencing analysis

Whole exome capture was performed with a SeqCap EZ Human Exome v2.0 kit (Roche NimbleGen, Madison, WI, USA), and sequencing was carried out on a HiSeq 2000 instrument (Illumina Inc, San Diego, CA, USA). Exome sequence alignment and variant calling were performed with DNAnexus software (DNAnexus Inc, Mountain View, CA, USA). Tumor-specific variants were identified based on a minimum variant allele ratio of 20%, a minimum read depth of 20, and absence of the variant in a matched normal specimen. Nucleotide variants were translated, and non-synonymous variants were identified using SIFT, PolyPhen2, Mutation Assessor, and SNPs&GO [53].

## SUPPLEMENTARY FIGURES AND TABLES





## References

[1] Hirota S, Isozaki K, Moriyama Y, Hashimoto K, Nishida T, Ishiguro S, Kawano K, Hanada M, Kurata A, Takeda M, Muhammad TG, Matsuzawa Y, Kanakura Y (1998). Gain-of-function mutations of c-kit in human gastrointestinal stromal tumors. Science.

[2] Heinrich MC, Corless CL, Duensing A, McGreevey L, Chen CJ, Joseph N, Singer S, Griffith DJ, Haley A, Town A, Demetri GD, Fletcher CD, Fletcher JA (2003). PDGFRA activating mutations in gastrointestinal stromal tumors. Science.

[3] Blanke CD, Rankin C, Demetri GD, Ryan CW, Von Mehren M, Benjamin RS, Raymond AK, Bramwell VH, Baker LH, Maki RG, Tanaka M, Hecht JR, Heinrich MC (2008). Phase III randomized, intergroup trial assessing imatinib mesylate at two dose levels in patients with unresectable or metastatic gastrointestinal stromal tumors expressing the kit receptor tyrosine kinase: S0033. J Clin Oncol.

[4] Bauer S, Hartmann JT, de Wit M, Lang H, Grabellus F, Antoch G, Niebel W, Erhard J, Ebeling P, Zeth M, Taeger G, Seeber S, Flasshove M (2005). Resection of residual disease in patients with metastatic gastrointestinal stromal tumors responding to treatment with imatinib. Int J Cancer.

[5] Wardelmann E, Thomas N, Merkelbach-Bruse S, Pauls K, Speidel N, Buttner R, Bihl H, Leutner CC, Heinicke T, Hohenberger P (2005). Acquired resistance to imatinib in gastrointestinal stromal tumours caused by multiple KIT mutations. Lancet Oncol.

[6] Heinrich MC, Corless CL, Blanke CD, Demetri GD, Joensuu H, Roberts PJ, Eisenberg BL, Von Mehren M, Fletcher CD, Sandau K, McDougall K, Ou W B, Chen CJ (2006). Molecular correlates of imatinib resistance in gastrointestinal stromal tumors. J Clin Oncol.

[7] Liu Y, Perdreau SA, Chatterjee P, Wang L, Kuan SF, Duensing A (2008). Imatinib mesylate induces quiescence in gastrointestinal stromal tumor cells through the CDH1-SKP2-p27Kip1 signaling axis. Cancer Res.

[8] Gupta A, Roy S, Lazar AJ, Wang WL, McAuliffe JC, Reynoso D, McMahon J, Taguchi T, Floris G, Debiec-Rychter M, Schoffski P, Trent JA, Debnath J (2010). Autophagy inhibition and antimalarials promote cell death in gastrointestinal stromal tumor (GIST). Proc Natl Acad Sci U S A.

[9] Hsueh YS, Chang HH, Chiang NJ, Yen CC, Li CF, Chen LT (2014). MTOR inhibition enhances NVP-AUY922-induced autophagy-mediated KIT degradation and cytotoxicity in imatinib-resistant gastrointestinal stromal tumors. Oncotarget.

[10] Deveraux QL, Takahashi R, Salvesen GS, Reed JC (1997). X-linked IAP is a direct inhibitor of cell-death proteases. Nature.

[11] Hingorani P, Dickman P, Garcia-Filion P, White-Collins A, Kolb EA, Azorsa DO (2013). BIRC5 expression is a poor prognostic marker in Ewing sarcoma. Pediatr Blood Cancer.

[12] Miura K, Fujibuchi W, Ishida K, Naitoh T, Ogawa H, Ando T, Yazaki N, Watanabe K, Haneda S, Shibata C, Sasaki I (2011). Inhibitor of apoptosis protein family as diagnostic markers and therapeutic targets of colorectal cancer. Surg Today.

[13] Tamm I, Kornblau SM, Segall H, Krajewski S, Welsh K, Kitada S, Scudiero DA, Tudor G, Qui YH, Monks A, Andreeff M, Reed JC (2000). Expression and prognostic significance of IAP-family genes in human cancers and myeloid leukemias. Clin Cancer Res.

[14] Aird KM, Ghanayem RB, Peplinski S, Lyerly HK, Devi GR (2010). X-linked inhibitor of apoptosis protein inhibits apoptosis in inflammatory breast cancer cells with acquired resistance to an ErbB1/2 tyrosine kinase inhibitor. Mol Cancer Ther.

[15] Small S, Keerthivasan G, Huang Z, Gurbuxani S, Crispino JD (2010). Overexpression of survivin initiates hematologic malignancies in vivo. Leukemia.

[16] Gyrd-Hansen M, Meier P (2010). IAPs: from caspase inhibitors to modulators of NF-kappaB, inflammation and cancer. Nat Rev Cancer.

[17] LaCasse EC, Mahoney DJ, Cheung HH, Plenchette S, Baird S, Korneluk RG (2008). IAP-targeted therapies for cancer. Oncogene.

[18] Tamm I, Wang Y, Sausville E, Scudiero DA, Vigna N, Oltersdorf T, Reed JC (1998). IAP-family protein survivin inhibits caspase activity and apoptosis induced by Fas (CD95), Bax, caspases, and anticancer drugs. Cancer Res.

[19] Marino-Enriquez A, Ou WB, Cowley G, Luo B, Jonker AH, Mayeda M, Okamoto M, Eilers G, Czaplinski JT, Sicinska E, Wang Y, Taguchi T, Demetri GD (2014). Genome-wide functional screening identifies CDC37 as a crucial HSP90-cofactor for KIT oncogenic expression in gastrointestinal stromal tumors. Oncogene.

[20] Xia M, Knezevic D, Vassilev LT (2011). p21 does not protect cancer cells from apoptosis induced by nongenotoxic p53 activation. Oncogene.

[21] Carter BZ, Mak DH, Schober WD, Koller E, Pinilla C, Vassilev LT, Reed JC, Andreeff M (2010). Simultaneous activation of p53 and inhibition of XIAP enhance the activation of apoptosis signaling pathways in AML. Blood.

[22] Cheng Q, Ling X, Haller A, Nakahara T, Yamanaka K, Kita A, Koutoku H, Takeuchi M, Brattain MG, Li F (2012). Suppression of survivin promoter activity by YM155 involves disruption of Sp1-DNA interaction in the survivin core promoter. Int J Biochem Mol Biol.

[23] Yamauchi T, Nakamura N, Hiramoto M, Yuri M, Yokota H, Naitou M, Takeuchi M, Yamanaka K, Kita A, Nakahara T, Kinoyama I, Matsuhisa A, Kaneko N (2012). Sepantronium bromide (YM155) induces disruption of the ILF3/p54(nrb) complex, which is required for survivin expression. Biochem Biophys Res Commun.

[24] Fulda S (2014). Molecular pathways: targeting inhibitor of apoptosis proteins in cancer—from molecular mechanism to therapeutic application. Clin Cancer Res.

[25] Ko TK, Chuah CT, Huang JW, Ng KP, Ong ST (2014). The BCL2 inhibitor ABT-199 significantly enhances imatinib-induced cell death in chronic myeloid leukemia progenitors. Oncotarget.

[26] Giagkousiklidis S, Vellanki SH, Debatin KM, Fulda S (2007). Sensitization of pancreatic carcinoma cells for gamma-irradiation-induced apoptosis by XIAP inhibition. Oncogene.

[27] Varfolomeev E, Vucic D (2008). (Un)expected roles of c-IAPs in apoptotic and NFkappaB signaling pathways. Cell Cycle.

[28] El Rifai W, Sarlomo-Rikala M, Andersson LC, Knuutila S, Miettinen M (2000). DNA sequence copy number changes in gastrointestinal stromal tumors: tumor progression and prognostic significance. Cancer Res.

[29] Islam A, Kageyama H, Takada N, Kawamoto T, Takayasu H, Isogai E, Ohira M, Hashizume K, Kobayashi H, Kaneko Y, Nakagawara A (2000). High expression of Survivin, mapped to 17q25, is significantly associated with poor prognostic factors and promotes cell survival in human neuroblastoma. Oncogene.

[30] Keats JJ, Fonseca R, Chesi M, Schop R, Baker A, Chng WJ, Van WS, Tiedemann R, Shi CX, Sebag M, Braggio E, Henry T, Zhu YX (2007). Promiscuous mutations activate the noncanonical NF-kappaB pathway in multiple myeloma. Cancer Cell.

[31] Choschzick M, Tabibzada AM, Gieseking F, Woelber L, Jaenicke F, Sauter G, Simon R (2012). BIRC2 amplification in squamous cell carcinomas of the uterine cervix. Virchows Arch.

[32] Xia M, Knezevic D, Vassilev LT (2011). p21 does not protect cancer cells from apoptosis induced by nongenotoxic p53 activation. Oncogene.

[33] Dan HC, Sun M, Kaneko S, Feldman RI, Nicosia SV, Wang HG, Tsang BK, Cheng JQ (2004). Akt phosphorylation and stabilization of X-linked inhibitor of apoptosis protein (XIAP). J Biol Chem.

[34] Shen Y, Ren X, Ding K, Zhang Z, Wang D, Pan J (2014). Antitumor activity of S116836, a novel tyrosine kinase inhibitor, against imatinib-resistant FIP1L1-PDGFRalpha-expressing cells. Oncotarget.

[35] Nakahara T, Kita A, Yamanaka K, Mori M, Amino N, Takeuchi M, Tominaga F, Hatakeyama S, Kinoyama I, Matsuhisa A, Kudoh M, Sasamata M (2007). YM155, a novel small-molecule survivin suppressant, induces regression of established human hormone-refractory prostate tumor xenografts. Cancer Res.

[36] Tolcher AW, Mita A, Lewis LD, Garrett CR, Till E, Daud AI, Patnaik A, Papadopoulos K, Takimoto C, Bartels P, Keating A, Antonia S (2008). Phase I and pharmacokinetic study of YM155, a small-molecule inhibitor of survivin. J Clin Oncol.

[37] Nakamura N, Yamauchi T, Hiramoto M, Yuri M, Naito M, Takeuchi M, Yamanaka K, Kita A, Nakahara T, Kinoyama I, Matsuhisa A, Kaneko N, Koutoku H (2012). Interleukin enhancer-binding factor 3/NF110 is a target of YM155, a suppressant of survivin. Mol Cell Proteomics.

[38] Tang H, Shao H, Yu C, Hou J (2011). Mcl-1 downregulation by YM155 contributes to its synergistic anti-tumor activities with ABT-263. Biochem Pharmacol.

[39] Na YS, Yang SJ, Kim SM, Jung KA, Moon JH, Shin JS, Yoon DH, Hong YS, Ryu MH, Lee JL, Lee JS, Kim TW (2012). YM155 induces EGFR suppression in pancreatic cancer cells. PLoS One.

[40] Glaros TG, Stockwin LH, Mullendore ME, Smith B, Morrison BL, Newton DL (2012). The “survivin suppressants” NSC 80467 and YM155 induce a DNA damage response. Cancer Chemother Pharmacol.

[41] Amaravadi RK, Senzer NN, Martin LP, Schilder RJ, LoRusso P, Papadopoulos KP, Weng DE, Graham M, Adjei AA (2013). A phase I study of birinapant (TL32711) combined with multiple chemotherapies evaluating tolerability and clinical activity for solid tumor patients. J Clin Oncol.

[42] Krepler C, Chunduru SK, Halloran MB, He X, Xiao M, Vultur A, Villanueva J, Mitsuuchi Y, Neiman EM, Benetatos C, Nathanson KL, Amaravadi RK, Pehamberger H (2013). The novel SMAC mimetic birinapant exhibits potent activity against human melanoma cells. Clin Cancer Res.

[43] Yuan Z, Syrkin G, Adem A, Geha R, Pastoriza J, Vrikshajanani C, Smith T, Quinn TJ, Alemu G, Cho H, Barrett CJ, Arap W, Pasqualini R (2013). Blockade of inhibitors of apoptosis (IAPs) in combination with tumor-targeted delivery of tumor necrosis factor-alpha leads to synergistic antitumor activity. Cancer Gene Ther.

[44] Delahaye NF, Rusakiewicz S, Martins I, Menard C, Roux S, Lyonnet L, Paul P, Sarabi M, Chaput N, Semeraro M, Minard-Colin V, Poirier-Colame V, Chaba K (2011). Alternatively spliced NKp30 isoforms affect the prognosis of gastrointestinal stromal tumors. Nat Med.

[45] Muhlenberg T, Zhang Y, Wagner AJ, Grabellus F, Bradner J, Taeger G, Lang H, Taguchi T, Schuler M, Fletcher JA, Bauer S (2009). Inhibitors of deacetylases suppress oncogenic KIT signaling, acetylate HSP90, and induce apoptosis in gastrointestinal stromal tumors. Cancer Res.

[46] Taguchi T, Sonobe H, Toyonaga S, Yamasaki I, Shuin T, Takano A, Araki K, Akimaru K, Yuri K (2002). Conventional and molecular cytogenetic characterization of a new human cell line, GIST-T1, established from gastrointestinal stromal tumor. Lab Invest.

[47] Edris B, Espinosa I, Muhlenberg T, Mikels A, Lee CH, Steigen SE, Zhu S, Montgomery KD, Lazar AJ, Lev D, Fletcher JA, Beck AH, West RB (2012). ROR2 is a novel prognostic biomarker and a potential therapeutic target in leiomyosarcoma and gastrointestinal stromal tumour. J Pathol.

[48] Vichai V, Kirtikara K (2006). Sulforhodamine B colorimetric assay for cytotoxicity screening. Nat Protoc.

[49] Duensing A, Medeiros F, McConarty B, Joseph NE, Panigrahy D, Singer S, Fletcher CD, Demetri GD, Fletcher JA (2004). Mechanisms of oncogenic KIT signal transduction in primary gastrointestinal stromal tumors (GISTs). Oncogene.

[50] Laemmli UK (1970). Cleavage of structural proteins during the assembly of the head of bacteriophage T4. Nature.

[51] Simon S, Grabellus F, Ferrera L, Galietta LJ, Schwindenhammer B, Muehlenberg T, Taeger G, Eilers G, Treckmann J, Breitenbuecher F, Schuler M, Taguchi T, Fletcher JA (2013). DOG1 regulates growth and IGFBP5 in gastrointestinal stromal tumors. Cancer Res.

[52] Astolfi A, Nannini M, Pantaleo MA, Di Battista M, Heinrich MC, Santini D, Catena F, Corless CL, Maleddu A, Saponara M, Lolli C, Di S V, Formica S (2010). A molecular portrait of gastrointestinal stromal tumors: an integrative analysis of gene expression profiling and high-resolution genomic copy number. Lab Invest.

[53] Falchook GS, Trent JC, Heinrich MC, Beadling C, Patterson J, Bastida CC, Blackman SC, Kurzrock R (2013). BRAF mutant gastrointestinal stromal tumor: first report of regression with BRAF inhibitor dabrafenib (GSK2118436) and whole exomic sequencing for analysis of acquired resistance. Oncotarget.

