# Supplementary material for: Inhibitor of Apoptosis Proteins (IAPs) are commonly dysregulated in GIST and can be pharmacologically targeted to enhance the pro-apoptotic activity of imatinib
Source: Oncotarget. 2016 May 4;7(27):41390–403. doi: 10.18632/oncotarget.9159 (PMC5173067; doi:10.18632/oncotarget.9159)
Supplement: Supplementary file 1 [file oncotarget-07-41390-s001.pdf]

SUPPLEMENTARY FIGURES AND TABLES

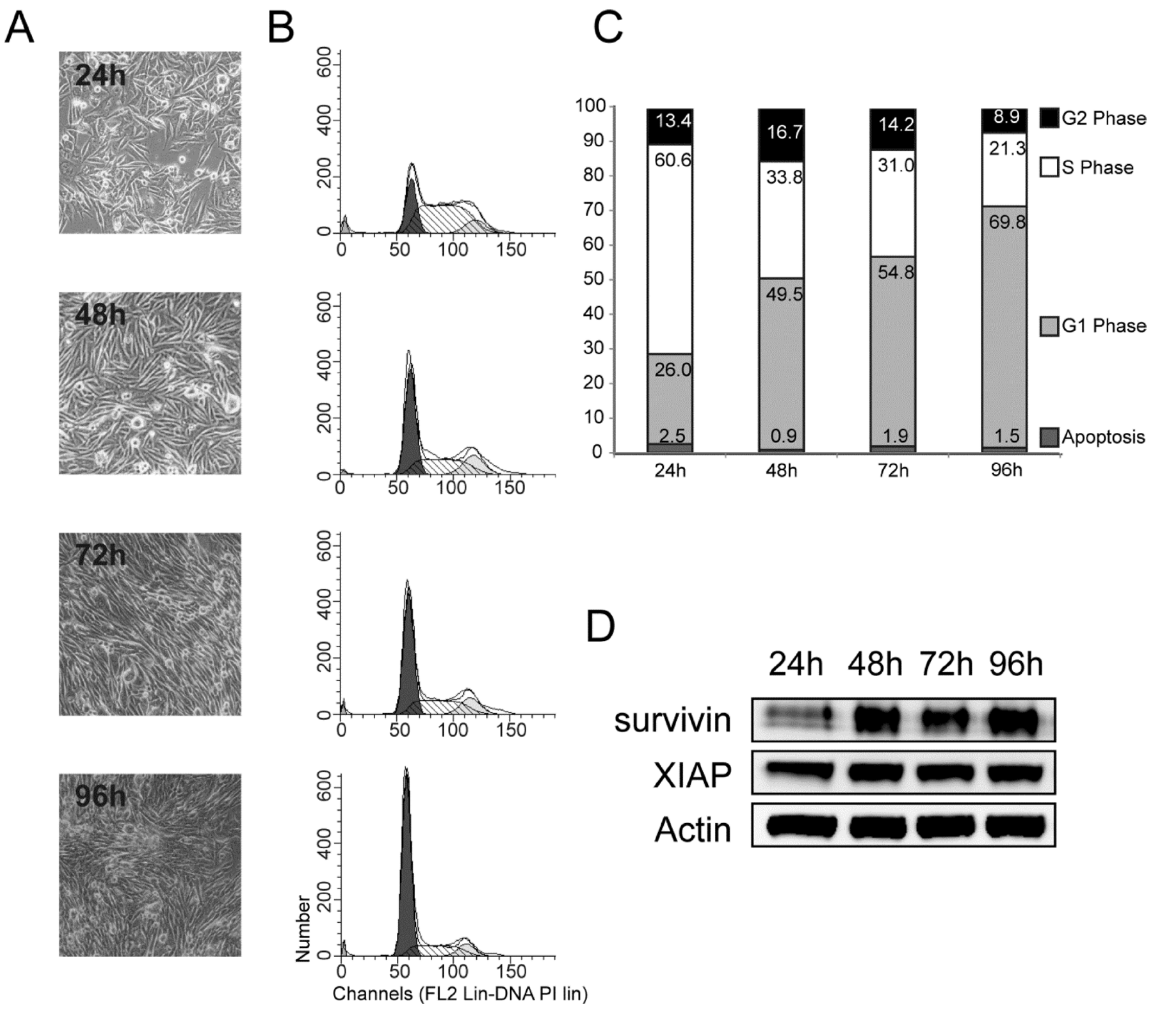

Supplementary Figure S1: Cell cycle distribution and survivin expression is influenced by cell density and the amount of apoptotic cells in the GIST-T1 cell line. Cells were seeded into 6-well-plates and analyzed 24h, 48h, 72h, and 96 hours after plating by photography, cell cycle analysis and Western Blot. **A.** Pictures of culture cells display increasing cell density. **B-C.** Flow cytometry for cell cycle analysis. Variable percentage of sub-G1/apoptotic cells, increasing G1, decreasing S-Phase population. **D.** Western Blot: stable expression of XIAP, variable expression of survivin contrary to amount of apoptosis/sub-G1-cells.

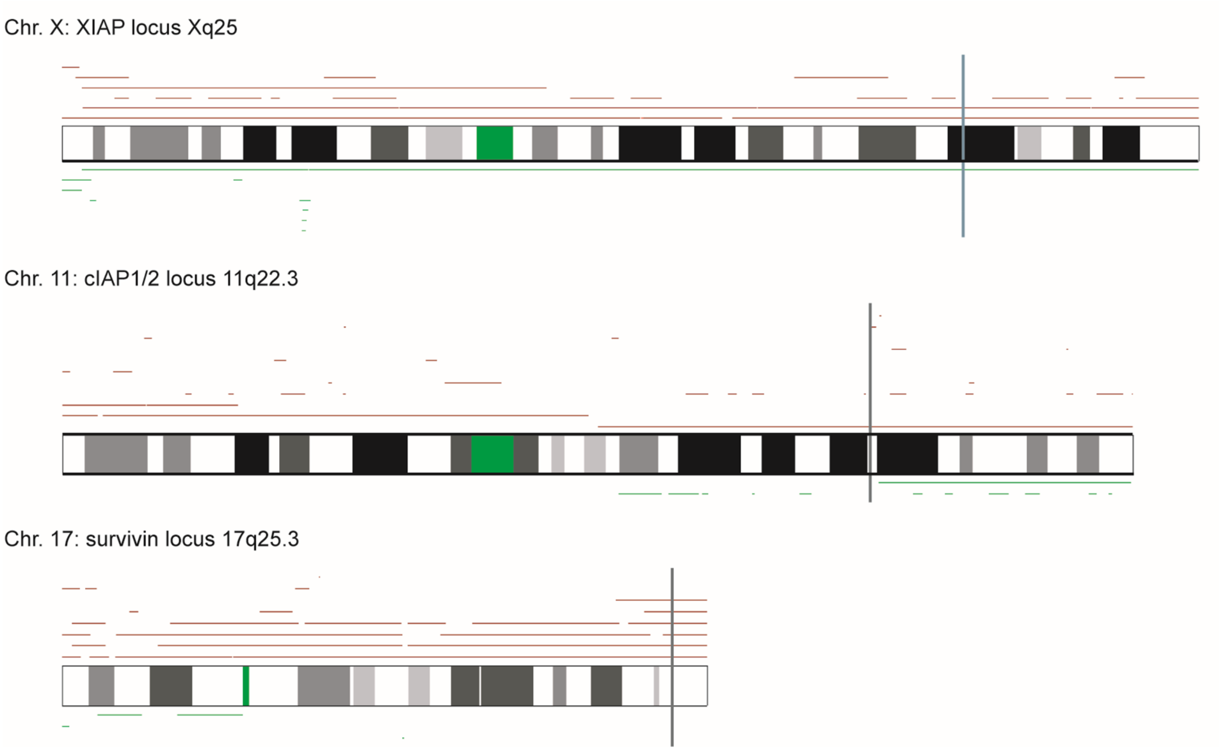

Supplementary Figure S2: IAP gene copy number alterations were found in a subset of GIST tumors. Schematic of copy number gains (above) and losses (beneath chromosome). IAP loci are marked in grey vertical lines.

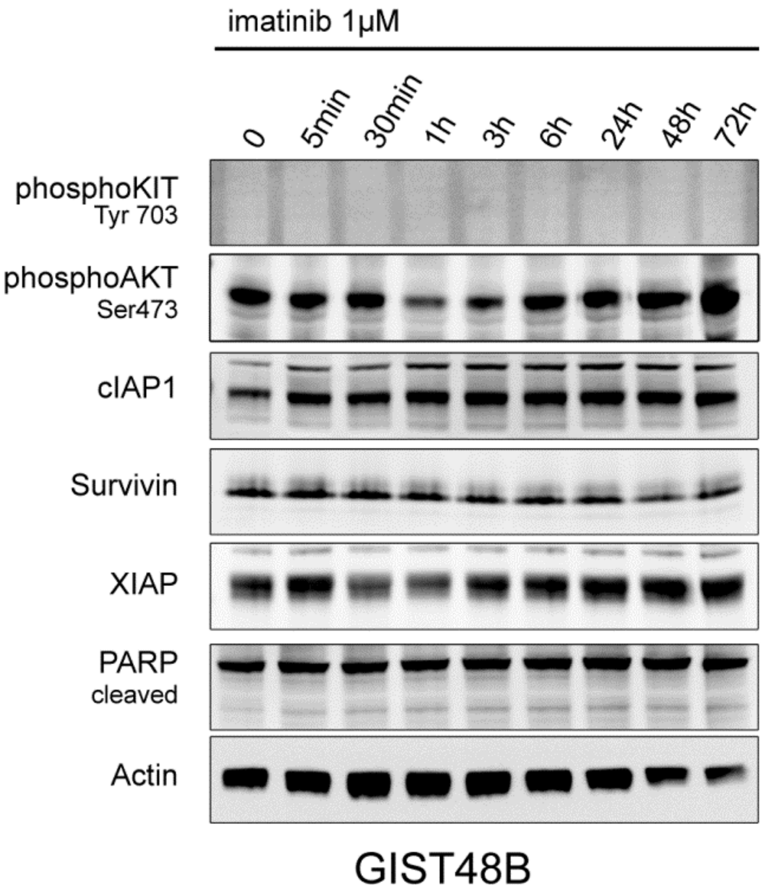

Supplementary Figure S3: KIT-negative cell line GIST48B was treated with imatinib to analyse unspecific cytotoxic effects. No differences were observed in IAP expression levels.

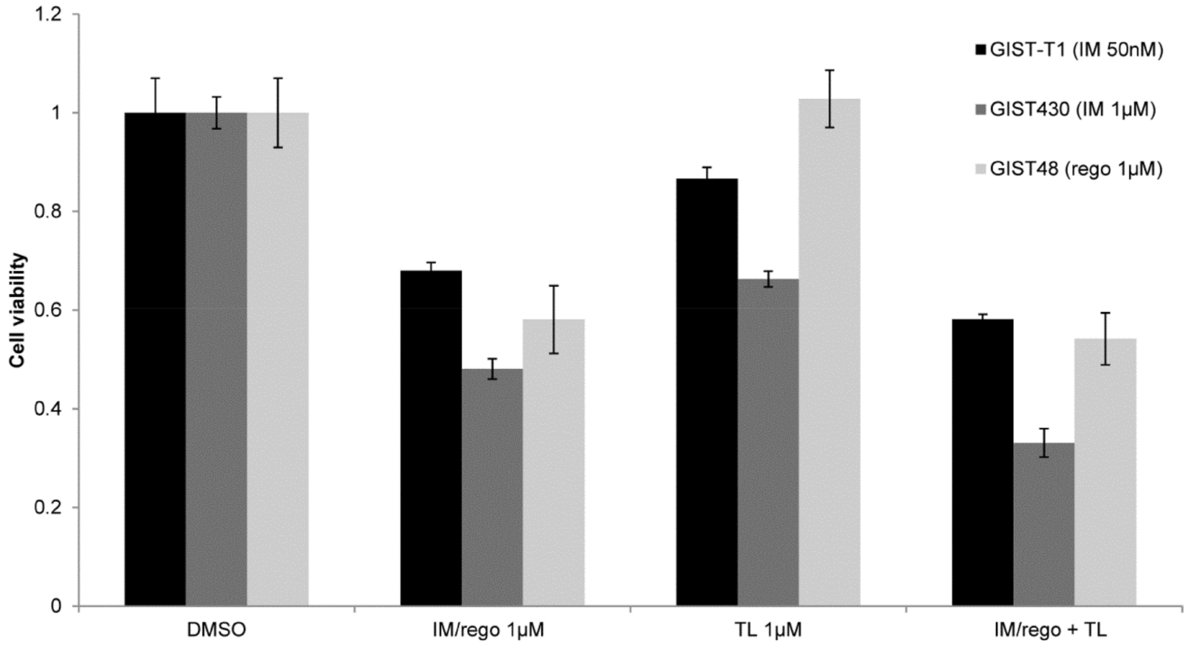

Supplementary Figure S4: Viability assays (SRB) were conducted after 3 days of treatment with TL and IM/regorafenib (rego) alone and combined TL+IM. The combination of TL and KIT inhibitors IM/regorafenib shows minor agonistic effects in GIST-T1, GIST430-11 and GIST48. Data are represented as mean +/- SEM.

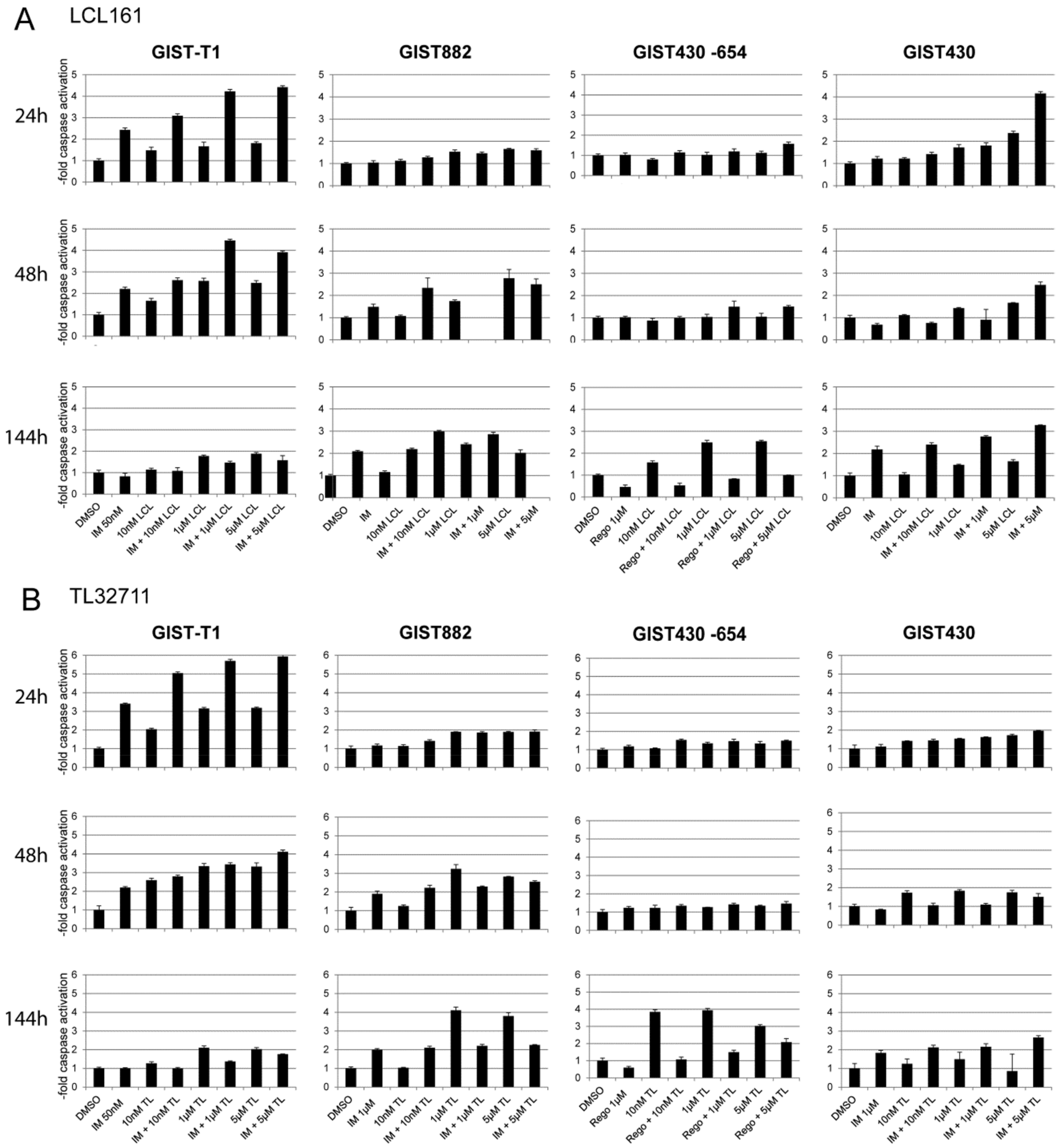

Supplementary Figure S5: Caspase Glo ® Caspase activation assays were conducted in GIST cell lines and apoptosis was measured after 24,48 and 144 hours of treatment with Smac mimetics alone and in combination with KIT inhibitors (A. LCL161, B. TL32711). In GIST-T1 and GIST430, agonistic proapoptotic effects could be observed.

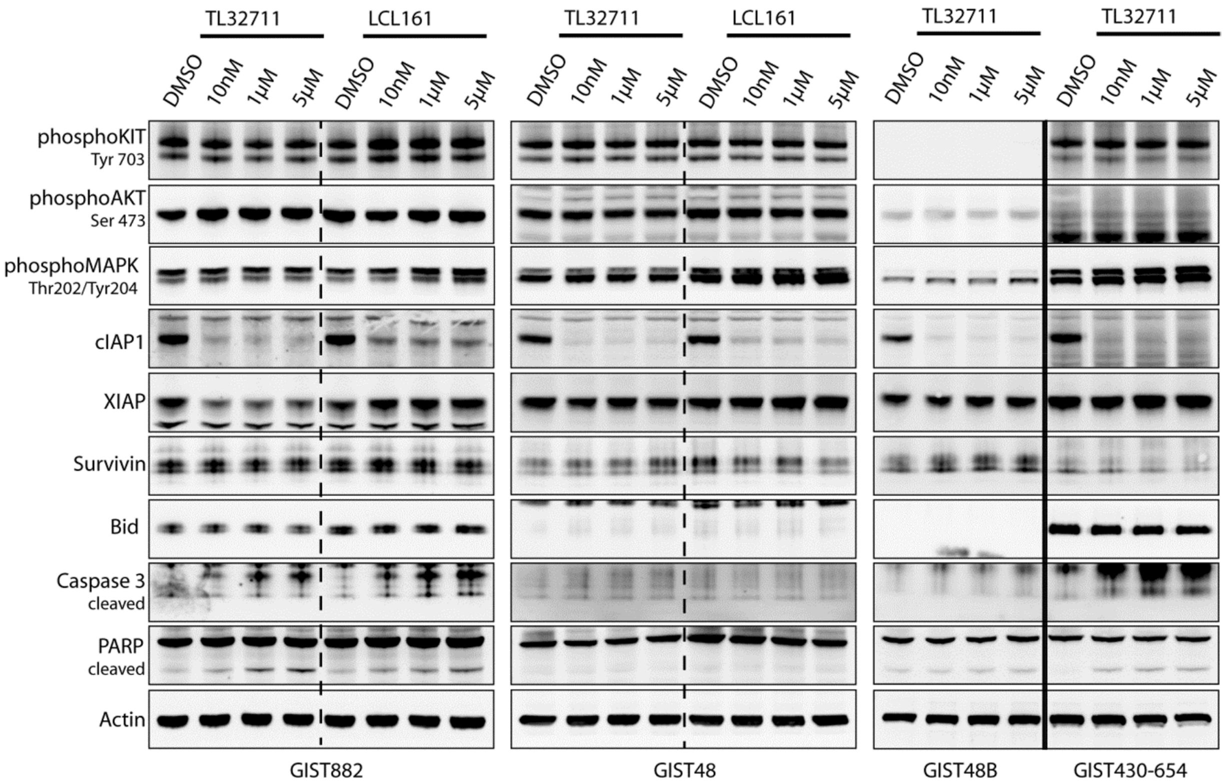

Supplementary Figure S6: Western Blot experiments after 24 hours of treatment with escalating doses of TL and LCL. Cellular IAP 1 was downregulated in all cell lines, whereas XIAP was only reduced in GIST882, treated with TL. Apoptosis was induced in GIST882 and GIST48B. The expression of pKIT, pAKT and survivin was not influenced.

Supplementary Table S1: Detailed information about patients referred to in Figure 1A

ID	gender	age	site	disease status at diagnosis	mutational status	cIAP	XIAP	survi-vin
1	m	73	small intestine	metastatic	KIT Exon 9 A502-Y503dup	yes	y	y
2	m	45	stomach	metastatic	KIT Exon 9 S476I	n	n	y
3	m	48	small intestine	metastatic	KIT Exon 11 Tyr553-Lys558del	y	y	y
4	m	66	rectum	localized	unknown	y	n	n
5	m	56	small intestine	unknown	KIT Ex.11 del	y	y	y
6	m	68	stomach	metastatic	(KIT Ex11/Ex17 Wildtyp, Ex9 / Ex13 unknown)	x	y	y
7	f	39	small intestine	metastatic	Exon 9 (dupl Codon 502 und 503)	y	n	y
8	m	72	rectum	metastatic	KIT Exon 11 V560D, Exon 17 C809V frameshift stopcodon 813	y	y	y
9,18	m	33	ileum	metastatic	KIT Exon 11 E554-V559del6, Exon 17 N822K	y	y	y
10	m	44	stomach, large intestine	metastatic	KIT in frame Deletion Exon 11 W557-K558del, Exon 13 V654A	n	y	y
11	m	67	small intestine	metastatic	KIT Exon 11 W557-K558del2, Exon 17 Y823D	y	n	y
12	m	56	liver metastasis	metastatic	unknown	y	y	y
13	m	56	unknown	unknown	unknown	y	y	n
14	m	46	jejunum	metastatic	KIT Exon 11 c.1653_1676del p.M552_V559del, Exon 14 c.2008_2009AC>GA; pT670E (gatekeeper-Mutation)	y	y	y
15	m	44	peritoneum	metastatic	wildtype	n	n	y
16	m	44	diaphragma	metastatic	unknown	y	y	y
17	f	40	little pelvis	metastatic	unknown	y	y	y
19	f	15	unknown	unknown	unknown	y	y	y
20	m	52	unknown	unknown	unknown	y	y	y

Supplementary Table S2: Detailed information about SNP array data

See Supplementary File 1

Supplementary Table S3: materials: antibodies, inhibitors and GIST cell lines

antibody	Product code	species	Distributor (Location)
XIAP	#2045	Rabbit	Cell Signaling (Beverly, MA, USA)
survivin	#2808	Rabbit	Cell Signaling (Beverly, MA, USA)
phospho-KIT (Tyr703/719)	#3073/#3391	Rabbit	Cell Signaling (Beverly, MA, USA)
phospho-AKT (Ser473)	#9271	Rabbit	Cell Signaling (Beverly, MA, USA)
cleaved Caspase 3	#9661	Rabbit	Cell Signaling (Beverly, MA, USA)
cleaved PARP	#9542	Rabbit	Cell Signaling (Beverly, MA, USA)
phospho-MAPK (Thr202/Tyr204)	#9101	Rabbit	Cell Signaling (Beverly, MA, USA)
MAPK	#9102	Rabbit	Cell Signaling (Beverly, MA, USA)
Actin (monoclonal, AC-15)	A5441	Mouse	Sigma-Aldrich (St. Louis, MA, USA)
KIT	A4502	Rabbit	DakoCytomation (Carpinterie, CA, USA)
P27/Kip1	610241	Rabbit	BD Transduction Laboratories
Bim	#2819	Rabbit	Cell Signaling (Beverly, MA, USA)
cIAP1 (polyclonal)	AF8181	Goat	R&D Biosciences
**Inhibitor**	**Target**	**Distributor(Location)**	
Imatinib (IM)	KIT/RTKs	LC Laboratories (Woburn, MA, USA)	
Regorafenib (Rego)	KIT/RTKs	Selleck Chemicals/Biozol (Eching, Germany)	
Sunitinib (SU)	KIT/RTKs	LC Laboratories (Woburn, MA, USA)	
YM155 (YM)	trancription factors of survivin	Selleck Chemicals/Biozol (Eching, Germany)	
TL32711/Birinapant (TL)	SMAC	Active BioChem (Wan Chai, HongKong, P.R. China)	
LCL161 (LCL)	SMAC	Novartis (Basel, Switzerland)	
**Cell line**	**IM sens./res.**	**KIT mutational status**	
GIST-T1	sensitive	KIT exon 11 57bp deletion	
GIST882	sensitive	KIT exon 13 activating mutation (K642E)	
GIST430	sensitive	KIT exon 11 heterozygous in frame deletion	
GIST48	resistant	KIT exon 11 homozygous mutation<br>KIT exon 17 heterozygous kinase-loop mutation (D820A)	
GIST48B	resistant	Subclone of GIST48, no detectable expression of KIT transcript or KIT protein	
