# Supplementary material for: Inhibitor of Apoptosis Proteins (IAPs) are commonly dysregulated in GIST and can be pharmacologically targeted to enhance the pro-apoptotic activity of imatinib
Source: Oncotarget. 2016 May 4;7(27):41390–403. doi: 10.18632/oncotarget.9159 (PMC5173067; doi:10.18632/oncotarget.9159)
Supplement: Supplementary file 2 [file oncotarget-07-41390-s002.docx]

**Suppl. Table S2:** Detailed information about SNP array data

| **ID** | **gen-der** | **age** | **site** | **disease status at diagnosis** | **mutational status** | **detail** |
| --- | --- | --- | --- | --- | --- | --- |
| GIST_02 _a_ | f | 85 | stomach | localized | KIT Ex.11 V560D | LOH 11q22.4 |
| GIST_04 _a_ | m | 79 | stomach | localized | KIT Ex. 9 AY502-503 insertion |  |
| GIST_05 _a_ | m | 68 | stomach | localized | PDGFr-a exon 12 SPDGHE566-571RIQ | amp Chr.17 |
| GIST_07_a_ | f | 28 | stomach | metastatic | KIT and PDGFr-a WT |  |
| GIST_08 _a_ | m | 62 | stomach | localized | KIT exon 11 V559D |  |
| GIST_09 _a_ | m | 54 | stomach | localized | KIT exon 11 insertion TQLPYDHKWEFP574-585 at P585 | LOH 11q22.4 |
| GIST_10 _a_ | m | 30 | stomach | metastatic | KIT and PDGFr-a WT |  |
| GIST_11 _a_ | m | 65 | stomach | localized | KIT exon 11 deletion WK557-558 |  |
| GIST_12 _a_ | f | 66 | stomach | localized | PDGFr-a exon 14 K646E |  |
| GIST_13 _a_ | m | 46 | small intestine | localized | KIT exon 11 V559D |  |
| GIST_14 _a_ | m | 56 | stomach | metastatic | KIT exon 11 homozygous deletion WK557-558 |  |
| GIST_15 _a_ | f | 64 | stomach | localized | PDGFr-a exon 18 del DIMH842-845 | XIAP locus only Xq25 |
| GIST_16 _a_ | f | 62 | stomach | localized | KIT exon 11 L576P | del 11q14.1-qter homo |
| GIST_17 _a_ | m | 37 | NA | metastatic | PDGFr-a exon 12 del SPDGHE566-571R | amp 17q25.1-qter |
| GIST_18 _a_ | m | NA | NA | NA | KIT Exon 11 V559G | del 11q21-qter homo, XIAP locus only Xq25amp |
| GIST_19 _a_ | m | 85 | stomach | metastatic | PDGFr-a exon 18 Y849C |  |
| GIST_20 _a_ | m | 38 | small intestine | metastatic | KIT exon 11 deletion MYEVQW552-557Z+KIT exon 18 point mutant A829P+SNP L862L |  |
| GIST_21 _a_ | f | 25 | stomach | NA | KIT and PDGFr-a WT |  |
| GIST_22 _a_ | f | 76 | stomach | NA | PDGFr-a exon 18 pm D842V | XIAP locus only Xq25amp, 17q25.3amp |
| GIST_23 _a_ | f | 47 | stomach | NA | KIT exon 11 V559D | XIAP locus only Xq25 amp. |
| GIST_24 _a_ | f | 18 | stomach | metastatic | KIT and PDGFr-a WT | XIAP locus only Xq25 amp. |
| GIST_25 _a_ | m | 84 | NA | NA | KIT del WKV557-559F | del11q22.1-22.2 homo (LOH) |
| GIST_26 _a_ | m | 49 | stomach | localized | PDGFr-a exon 12 V561D |  |
| GIST_27 _a_ | m | 52 | NA | NA | KIT exon 11 del KV558-559N |  |
| GIST_28 _a_ | f | 87 | NA | NA | KIT exon 11 W557G |  |
| **Essen_1** | **f** | **70** | **NA** | **metastatic** | **KIT and PDGFr-a WT (NF-1)** |  |
| **Essen_2** | **f** | **41** | **peritoneum** | **metastatic** | **KIT exon 11 W557-K558del** |  |
| **Essen_3** | **m** | **35** | **epigastric** | **metastatic** | **KIT exon 11 E554-V559del6** | **17q24.3-qter amp.** |
| **Essen_4** | **m** | **72** | **cervix** | **metastatic** | **KIT exon 11 V560D** | **amp 11q13.2-qter** |
| **Essen_5** | **m** | **66** | **rectum** | **localized** | **KIT exon 11 V559D** |  |
| **Essen_6** | **m** | **45** | **stomach** | **metastatic** | **KIT exon 9 S476I** | **17 amp** |
| **Essen_7** | **m** | **68** | **stomach** | **metastatic** | **KIT exon 11/exon 17 WT** |  |
| **Essen_8** | **m** | **56** | **jejunum** | **localized** | **exon 11 deletion PDGFR Exon 18 mut.** |  |
| **Essen_9** | **f** | **39** | **small intestine** | **metastatic** | **KIT Exon9 (dup Codon 502 und 503) Exon 17 Y823** | **Xq24-qter amp** |
| **Essen_10** | **m** | **48** | **jejunum** | **metastatic** | **KIT Exon 11 Y553_K558del** | **Xq21.1-qter amp 11q22.2 amp** |
| **Essen_11** | **m** | **76** | **stomach** | **metastatic** | **KIT Exon 11 6bp del** |  |
| **Essen_12** | **m** | **56** | **stomach** | **metastatic** | **NA** |  |
| **Essen_13** | **m** | **65** | **liver** | **metastatic** | **KIT Exon 11 W557-V559 del** | **17q25.3 - qter amp** |

cIAP amp: amplified; LOH: loss of heterozygosity; homo: homozygous; mut: mutated; dup: duplicaded

cIAP locus (11q22); XIAP locus (Xq25); survivin locus (17q25)

_a_ Data from GEO (GSE 20709, see Material and Methods section)
